# Potential Osteoinductive Effects of Calcitriol on the m-RNA of Mesenchymal Stem Cells Derived from Human Alveolar Periosteum

**DOI:** 10.1155/2016/3529561

**Published:** 2016-12-25

**Authors:** Hsiang-Hsi Hong, Adrienne Hong, Tzung-Hai Yen, Yen-Li Wang

**Affiliations:** ^1^Department of Periodontics, Chang Gung Memorial Hospital, Linkou, Taiwan; ^2^Chang Gung University, Taoyuan, Taiwan; ^3^School of Dental Technology, College of Oral Medicine, Taipei Medical University, Taipei, Taiwan; ^4^California Northstate University, College of Medicine, Elk Grove, CA, USA; ^5^Division of Clinical Toxicology, Department of Nephrology, Chang Gung Memorial Hospital, Linkou, Taiwan; ^6^Kidney Research Center, Chang Gung Memorial Hospital, Linkou, Taiwan; ^7^Center for Tissue Engineering, Chang Gung Memorial Hospital, Linkou, Taiwan; ^8^Department of Periodontics, Chang Gung Memorial Hospital, Taoyuan, Taiwan

## Abstract

This study characterized alveolar periosteum-derived mesenchymal stem cells (P-MSCs) and examined the hypothesis that 1,25-(OH)_2_D_3_ (calcitriol) exerts osteoinductive effects on P-MSCs. The mRNA expressions of alkaline phosphatase (ALP), bone sialoprotein (BSP), core-binding factor alpha-1 (CBFA1), collagen-1 (Col-1), osteocalcin (OCN), and vitamin D_3_ receptor (VDR) were assessed after incubation with calcitriol for 2 weeks. Vitamin C as positive control (Vit. C-p) increased ALP and CBFA1 mRNA expression at both 1 and 2 weeks and increased BSP and Col-1 mRNA expression only at the first week. A concentration of 10^−8^ M calcitriol enhanced ALP, CBFA1, Col-1, and OCN mRNA expression at both weeks and BSP mRNA expression at the first week. Furthermore, 10^−7^ M calcitriol increased the mRNA expressions of all compounds at both weeks, except that of CBFA1 at the first week. 10^−8^ M calcitriol and Vit. C-p enhanced ALP activity at the second and third weeks. The results revealed that 10^−9^, 10^−8^, and 10^−7^ M calcitriol induced osteoinduction in alveolar P-MSCs by increasing ALP, CBFA1, Col-1, and OCN mRNA expression. A 10^−7^ M calcitriol yielded a higher mRNA expression than Vit. Cp on VDR and OCN mRNA expression at both weeks and on Col-1 mRNA at the second week.

## 1. Introduction

Mesenchymal stem cells (MSCs) can be isolated from various tissues, including long bone periosteum and dental tissues [1]. To date, six types of human dental stem cells, namely, stem cells from postnatal dental pulp [2], exfoliated deciduous teeth [3], periodontal ligament [4], apical papilla [5], dental follicle [6], and gingival tissue [7], have been isolated from and characterized with the parent tissues. The cambium layer of the periosteum primarily contains a mixed cell population of fibroblasts [8], osteoblasts [9], and pericytes [10] and a critical subpopulation identified as MSCs [11]. The accessibility of the periosteum and favorable bone regeneration results have been reported [12], and an increasing number of dentists are applying alveolar bone regeneration in periodontology and implant dentistry. However, few studies have explored the regenerative potential of alveolar periosteum MSCs (P-MSCs) in dentistry.

Vitamin D, a group of fat-soluble secosteroids, is crucial for enhancing the intestinal absorption of calcium, iron, magnesium, phosphate, and zinc. Calcitriol [1,25-(OH)_2_D_3_; Vit. D_3_] circulates as a hormone in the blood, adjusts calcium and phosphate concentrations in the circulatory system, and supports remodeling and healthy bone growth. Furthermore, calcitriol modifies neuromuscular and immune functions [13]. Low Vit. D_3_ levels are associated with skeletal fragility and fractures; Vit. D_3 _metabolites are major contributors of bone and mineral homeostasis, including the effect of 24R,25-(OH)_2_D_3 _on the osteoblastic differentiation of human MSCs [14]. Bone homeostasis and repair are regulated by a network of Vit. D_3 _metabolites. Calcitriol, the most biologically active form of Vit. D_3_, serves various physiological functions in the body, the most crucial being the maintenance of the calcium and phosphorus balance, which affects bone health. In addition, Vit. D_3_ is essential for calcification [15]. In a study on 2-day-old Kunming white mice, Vit. C-p and Vit. D_3_ supplements effectively promoted osteoblast differentiation in mouse embryoid bodies [16]. Moreover, calcitriol stimulates the in vitro differentiation of human MSCs into osteoblasts, which can be monitored on the basis of the increase in the alkaline phosphatase (ALP) enzyme activity or osteocalcin (OCN) gene expression [14]. Few studies have investigated the dose–response and dose–time effects of calcitriol on human P-MSCs for bone tissue engineering.

The present study examined the effects of calcitriol-induced osteogenic differentiation on human alveolar periosteum-derived MSCs (P-MSCs) and assessed the optimal calcitriol concentration and incubation period required for osteogenic induction.

## 2. Materials and Methods

### 2.1. Tissue Preparation

Periosteal tissues were harvested from 12 male and 18 female patients under 65 years with a mean age of 48.1 ± 12.3 during surgery at the dental department of Chang Gung Memorial Hospital between 2011 and 2013. The Research and Ethics Committee of Chang Gung Memorial Hospital approved the study protocol. All patients provided written informed consent.

The harvested periosteal tissues were processed and stored in Dulbecco's phosphate-buffered saline (DPBS; Gibco, Carlsbad, CA, USA) with 300 U/mL of penicillin and 300 *μ*g/mL of streptomycin (Gibco BRL) and transferred to a laboratory within 4 hours, where the tissues were manually minced using scalpels. The obtained fragments were placed on 35-mm culture plates (Corning) containing 1.5 mL of growth medium 1 (*α*-modified Eagle's medium [HyClone], 10% fetal bovine serum [FBS, Invitrogen], 300 U/mL of penicillin, and 300 *μ*g/mL of streptomycin). When these periosteum-derived cells (PDCs) reached subconfluence (80%), the adherent cells were detached and separated. Another growth medium (*α*-modified Eagle's medium, 10% FBS, 100 U/mL of penicillin, and 100 *μ*g/mL of streptomycin) was applied for cell culture. The first replanted cells were labeled P-MSC passage 1. Subsequent passages of cultured P-MSCs were achieved by following the same treatment protocol when the previous passages reached 80% confluence.

All assays were performed using P-MSC passages 3 to 5. The induced osteogenic, chondrogenic, and adipogenic differentiation were evaluated through culture staining.

### 2.2. Flow Cytometry

In this experiment, 1 × 10^6^ cells were harvested through 0.25% trypsin digestion and collected through centrifugation. Cell pellets were washed three times using 1x DPBS, following which 1x permeabilization buffer (eBioscience, San Diego, CA) was added. This buffer was used to fix and permeabilize cells prior to the intracellular staining of cytokines and other cytoplasmic and nuclear antigens. Thereafter, surface marker antibodies against CD19 (FITC, BD), CD34 (FITC, BD), CD44 (PE, BD), CD45 (FITC, BD), CD73 (PE, BD), CD90 (APC, BD), STRO-1 (Alex, BioLegend), and HLA-DR (FITC, BD) were added to the cells and incubated for 30 minutes at 4°C in the dark. The cells were washed twice using 1x DPBS, fixed using 2% formaldehyde, and analyzed using a FACSVerse™ flow cytometer (BD Biosciences) that measured 10,000–20,000 cells per sample.

### 2.3. Cell Attachment and Viability during Osteogenic, Adipogenic, and Chondrogenic Differentiation

P-MSC samples were independently induced for differentiation into osteogenic, adipogenic, and chondrogenic lineages. Cells from passages 3–5 were cultured on six-well plates with specific differentiation media containing differentiation-inducing reagents.

For osteogenic differentiation, the cells were cultured in *α*-minimum essential medium (MEM) at a density of 5000 cells per well on six-well plates. On achieving approximately 80% confluence, the cells were cultured in an osteogenic medium containing *α*-MEM (HyClone), 5% FBS (Invitrogen), *β*-glycerophosphate (10 mM; Sigma), dexamethasone (100 nM; Sigma), and 2-phospho-L-ascorbic acid trisodium salt (Vit. C-p; 100 *μ*M; Sigma). The media were changed twice per week. To assess their potential to differentiate into the osteogenic lineage, the cells were stained using von Kossa, which distinguishes calcified deposits present in a culture.

For chondrogenic differentiation, the cells were cultured at a density of 5000 cells per well on six-well plates in a differentiation medium containing *α*-MEM (HyClone), 5% FBS (Invitrogen), dexamethasone (100 nM; Sigma), sodium pyruvate (100 *μ*g/mL; Gibco), insulin–transferrin–selenium-A (1x ITS, 1-1884; Sigma), transforming growth factor-*β* (10 ng/mL; R&D), and 5 mL of 2-phospho-L-ascorbic acid trisodium salt (Vit. C-p; 100 *μ*M; Sigma). Furthermore, the differentiation was evaluated according to the stained glycosaminoglycan in the culture identified through Alcian blue staining. For every cell sample subjected to differentiation, a control culture of PDCs was maintained in *α*-MEM and 5% FBS for 4-5 weeks and subjected to the same staining tests as those for the experimental cells.

For adipogenic differentiation, the cells were cultured in *α*-MEM at a density of 5000 cells per well on six-well plates. On achieving confluence, the cells were cultured in a media containing *α*-MEM (HyClone), 5% FBS (Invitrogen), 0.5 mM 3-isobutyl-1-methylxanthine (Sigma), dexamethasone (1 *μ*M; Sigma), insulin (5 *μ*g/mL; Sigma), and indomethacin (60 *μ*M; Sigma). The presence of lipid droplets was evaluated using Oil Red O staining to determine whether cells were differentiated into the adipogenic lineage.

### 2.4. Effects of Calcitriol on Osteogenesis

Passages 3–5 of P-MSCs were categorized into six groups according to the culture media: the control group [*α*-MEM (HyClone), 5% FBS (Invitrogen), 10 mM of *β*-glycerophosphate (Sigma), and 10^−7^ M dexamethasone (Sigma)], Vit. C-p group (control with 100 *μ*M Vit. C-p), 10^−10^ M calcitriol (Nang Kuang Pharmaceutical Co., Ltd) group (control with 10^−10^ M calcitriol), 10^−9^ M calcitriol group (control with 10^−9^ M calcitriol), 10^−8^ M calcitriol group (control with 10^−8^ M calcitriol), and 10^−7^ M calcitriol group (control with 10^−7^ M calcitriol).

### 2.5. Reverse Transcription and Quantitative Real-Time Polymerase Chain Reaction

After 7 days of osteoblast differentiation, the Trizol reagent was used for isolating total RNA, 1.0 *μ*g of which was reverse-transcribed using avian myeloblastosis virus reverse transcriptase (Roche).

First-strand complementary DNA (cDNA) was synthesized, and quantitative polymerase chain reaction (qPCR) was performed using 5 ng/*μ*L of cDNA. Quantitative real-time (qRT)-PCR was conducted using primers for ALP, BSP, CBFA1, Col-1, OCN, and VDR. To avoid DNA contamination by signals, forward and reverse sequences of each primer were designed on distinct exons; qPCR was performed using the SYBR Green PCR Master Mix and TaqMan Master Mix (Applied Biosystems) according to manufacturer instructions. Furthermore, the reactions were performed using the ViiA7 Real-Time PCR system (Applied Biosystems) with the TaqMan Master Mix at 50°C for 2 min, followed by 95°C for 10 min, and then 40 cycles each at 95°C for 15 s and 60°C for 60 s. The SYBR use was followed by PCR at 95°C for 10 min and then 40 cycles each at 95°C for 15 s, 60°C for 60 s, and 60°C for 15 min. The Ct values for ALP, BSP, CBFA1, Col-1, VDR, and OCN messenger RNAs (mRNAs) were normalized to the value of the housekeeping gene GAPDH mRNA.

### 2.6. Effects of Vit. C-p and Varying Calcitriol Concentrations on Osteogenesis

To confirm the differentiation of human PDCs into a P-MSC-related osteoblast phenotype at the molecular level, we monitored the expression of human ALP, BSP, CBFA1, Col-1, OCN, and VDR mRNAs at the first and second weeks through qRT-PCR by using human-specific primers. The results are presented as the fold change relative to the control group results, which was set to a value of 1.

### 2.7. Primer Pairs Used for qRT-PCR Analysis

The primer pair sequences used are mentioned in Table 1.

### 2.8. Alkaline Phosphatase Activity

Osteogenesis was quantitated by measuring ALP activity. Passages 3–5 P-MSCs were grown in the osteogenic medium; the cultured cells were using 100 mM Tris and 1% Triton-100. Furthermore, the Alkaline Phosphatase Activity Colorimetric Assay Kit (BioVision) was used for determining the ALP activity at the second and third week of culture by using a previously described method [17]. This method employs the conversion of* p*-nitrophenyl phosphate to* p*-nitrophenol, determined at a wavelength of 450 nm, and the ALP activity is calculated from a standard value. In this study, the total* p*-nitrophenol formed was normalized to total protein in 1 hour to determine the ALP activity (ALP activity/min/mg protein). The fold change activity in the experimental and control groups at the second and third week of culture was also compared.

Because the ALP mRNA test revealed that P-MSCs significantly induced osteoblast differentiation at calcitriol concentrations of 10^−8^ M and 10^−7^ M, the ALP activity assay was attempted using these concentrations. The ALP activity of the control, Vit. C-p, 10^−8^ M calcitriol, and 10^−7^ M calcitriol groups was assessed at the second and third weeks of culture.

### 2.9. Statistical Analysis

All values are represented as means ± standard deviation (SD). Statistical significance was determined using an unpaired Student's *t*-test, and *P* < 0.05 was considered statistically significant.

## 3. Results

### 3.1. Cell Isolation, Morphology and Osteogenic, Chondrogenic, and Adipogenic Differentiation

**Figure 1:**
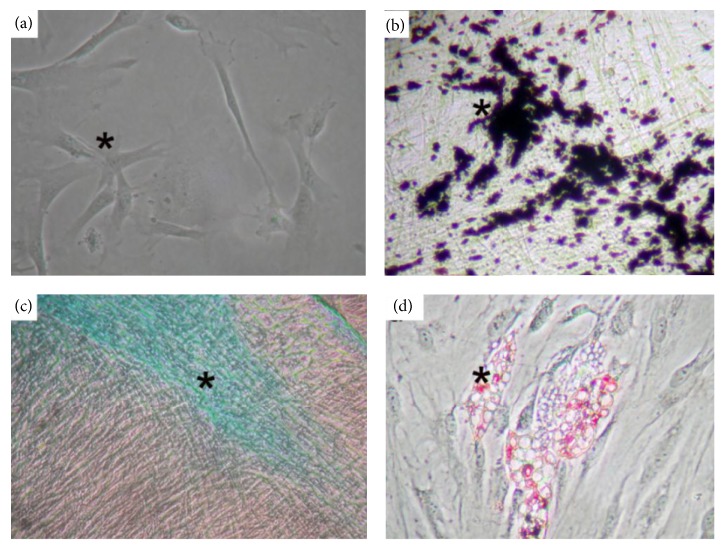
Differentiation study. (a) Mesenchymal stem cells (MSCs) were spindle-shaped with irregular processes and firmly attached to the culture plate after 1–3 days of primary culture (asterisk). Under in vitro culture conditions, MSCs were subpassaged and differentiated into (b) osteoblasts (asterisk, black, von Kossa staining), (c) chondrocytes (asterisk, blue, Alcian blue staining), and (d) adipocytes (asterisk, pink, Oil Red O staining).

Eventually, we examined P-MSCs from two male and three female patients. Under an optical microscope, the initial culture had small and round cells before attachment and spindle-shaped cells thereafter. PDCs proliferated in spherical colonies on days 1–14. Morphologically, phase-contrast microscopy revealed that most cells were spindle-shaped and that they formed a homogenous cell population (Figure 1). After the first passage, the cells no longer proliferated in clusters but exhibited a uniformly widespread pattern. PDCs were spindle-shaped with irregular processes and firmly attached themselves to the culture plate at day 3 of the primary culture (Figure 1(a)). Thereafter, the cells exhibited a homogeneous, fibroblast-like spindle shape (Figure 1).

At the end of the osteogenic differentiation, von Kossa staining revealed the presence of calcium deposits and osteogenic differentiation for 4–6 weeks (Figure 1(b)). Proteoglycan production, an indicator of chondrogenic differentiation, was observed by staining PDCs with Alcian blue (Figure 1(c)) for 4–6 weeks. For inducing adipogenic differentiation, PDCs were cultured in a specific medium, as previously described [7]. After 8 weeks of induction, PDC-derived adipocytes were stained using Oil Red O to confirm the presence of intracellular lipids (Figure 1(d)). The results revealed that PDCs could be decent subpassaged and potentially differentiated into P-MSCs.

### 3.2. Flow Cytometric Surface Marker Expression Analysis for PDCs

**Figure 2:**
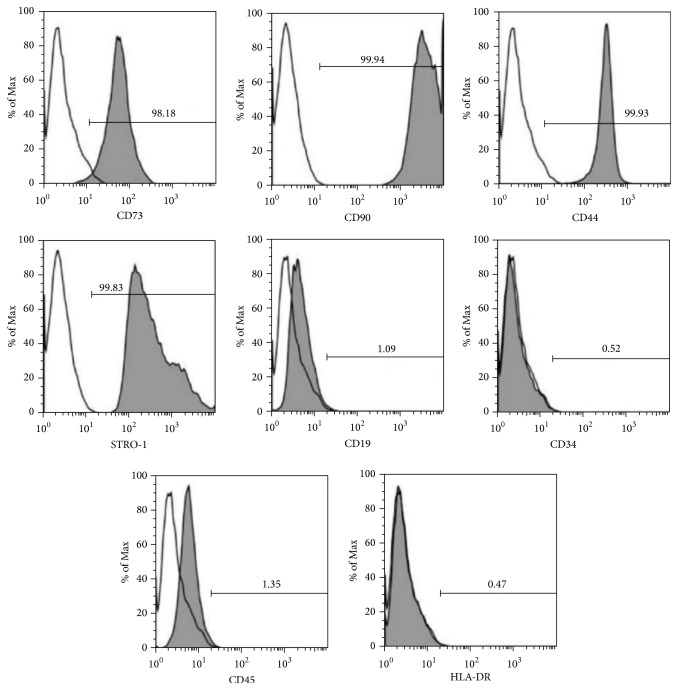
Surface marker expression of mesenchymal stem cells through flow cytometry. The surface markers expressed by mesenchymal stem cells mainly included CD73 (98.18%), CD90 (99.94%), STRO-1 (99.83%), and CD44 (99.93%) but not CD45 (1.35%), CD34 (0.52%), CD19 (1.09%), or HLA-DR (0.47%).

A flow cytometric assay was conducted for defining the surface marker expression of MSCs before seeding them into biomaterial scaffolds. The PDCs tested positive for the MSC markers CD73 (98.18%), CD90 (99.94%), STRO-1 (99.83%), and CD44 (99.93%) but negative for the hematopoietic lineage markers CD19 (1.09%), CD34 (0.52%), CD45 (1.35%), and HLA-DR (0.47%) (Figure 2).

### 3.3. Alkaline Phosphatase mRNA Expression at the First and Second Weeks

ALP mRNA, a known marker for detecting early osteogenic cell differentiation, acts as an ectoenzyme in the degradation of inorganic pyrophosphate for releasing phosphate for mineralization [18]. Compared with the negative control group, the fold changes of ALP mRNA expression of the first- and second-week cultures were 3.68 ± 2.24 and 5.12 ± 3.34 for the Vit. C-p group, 0.93 ± 0.14 and 0.95 ± 0.21 for the 10^−10^ M calcitriol group, 1.59 ± 0.41 and 1.93 ± 0.61 for the 10^−9^ M calcitriol group, 4.50 ± 1.69 and 4.82 ± 2.44 for the 10^−8^ M calcitriol group, and 1.49 ± 0.37 and 4.88 ± 1.61 for the 10^−7^ M calcitriol group, respectively (Figure 1(a)). The 10^−9^, 10^−8^, and 10^−7^ M calcitriol and Vit. C-p groups revealed significantly different ALP mRNA expression at both weeks (*P* < 0.05; Figure 1(a)). Data pooled and analyzed at 2 weeks revealed a similar developing pattern.

**Figure 3:**
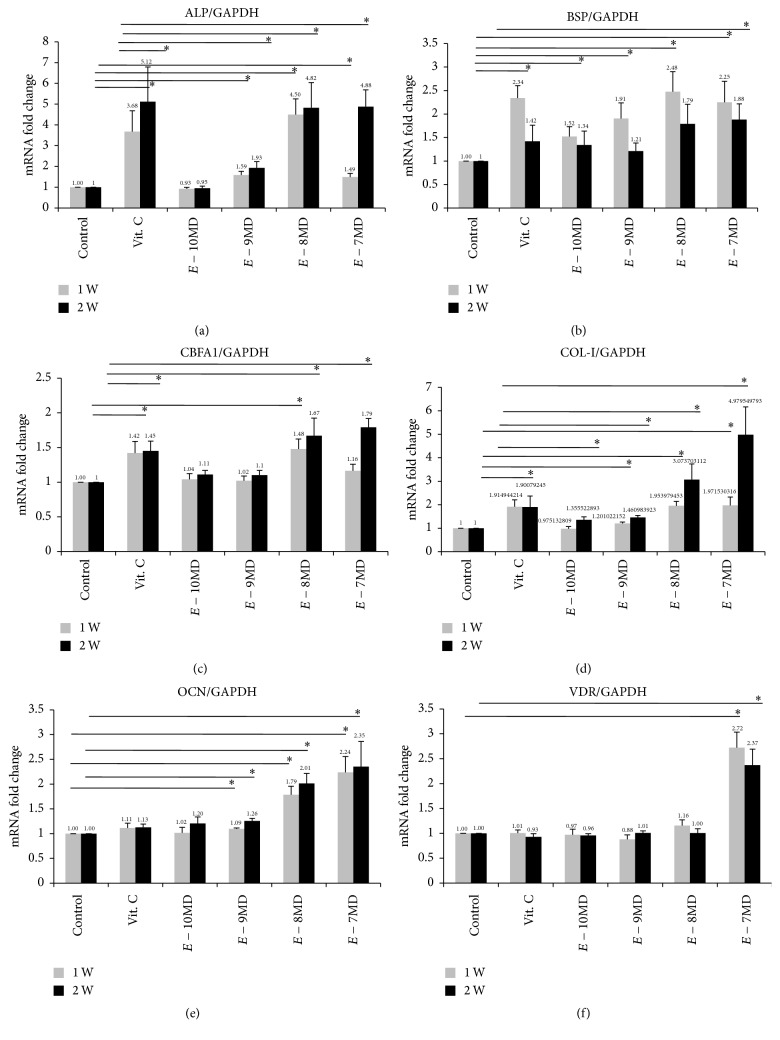
mRNA expressions of osteoblast differentiation for first and second weeks. (a) ALP expression, (b) BSP, (c) CBFA1, (d) COL-I, (e) OCN, and (f) VDR.

The 10^−9^, 10^−8^, and 10^−7^ M calcitriol groups revealed significant changes in ALP mRNA expression fold changes compared with the 10^−10^ M calcitriol group at the end of both weeks (*P* < 0.05). The Vit. C-p and 10^−7^and 10^−8^ M calcitriol groups revealed a similar ALP mRNA expression at both weeks; however, the 10^−7^ M and 10^−8^ M calcitriol groups revealed a significant variation at the first week (Figure 3(a) and Supplemental Table 1 available in Supplementary Material at http://dx.doi.org/10.1155/2016/3529561).

### 3.4. Bone Sialoprotein mRNA Expression at the First and Second Week

The fold changes of BSP mRNA expression between the first and second weeks generally revealed a nonsignificant change for all tested groups (Table 2). However, the fold changes of BSP mRNA expression mainly occurred at the first week; they were 2.34 ± 0.59, 1.52 ± 0.46, 1.91 ± 0.74, 2.48 ± 0.95, and 2.25 ± 1.00 for the Vit. C-p and 10^−10^ M, 10^−9^ M, 10^−8^ M, and 10^−7^ M calcitriol groups (Supplemental Table 2). At the second week, only the 10^−7^ M calcitriol group exhibited significantly higher BSP mRNA expression than did the control group (*P* < 0.05, Figure 3(b)). Nonsignificant differences were observed between the Vit. C-p and calcitriol groups (Supplemental Table 2).

### 3.5. Core-Binding Factor Alpha-1 mRNA Expression at the First and Second Weeks

The fold changes of CBFA1 mRNA expression between the first and second weeks were nonsignificant for most tested groups, except for the 10^−7^ M calcitriol subgroups (*P* = 0.009, Table 2). Vit. C-p and 10^−8^ M calcitriol significantly upregulated the CBFA1 mRNA expression in cells at both weeks compared with the expression in controls; by contrast, 10^−7^ M calcitriol upregulated the CBFA1 mRNA expression only at the second week. Differences among the three subgroups were nonsignificant (Figure 3(c) and Supplemental Table 3).

### 3.6. Collagen-1 mRNA Expression at the First and Second Weeks

The 10^−10^ and 10^−9^ M calcitriol groups exhibited slightly significant differences between the first and second weeks (Table 2). Compared with the control groups, Vit. C-p and 10^−9^, 10^−8^, and 10^−7^ M calcitriol at the first week and 10^−10^, 10^−9^, 10^−8^, and 10^−7^ M calcitriol at the second week significantly increased the Col-1 mRNA expression (Figure 3(d) and Supplemental Table 4).

### 3.7. Osteocalcin mRNA Expression at the First and Second Weeks

OCN mRNA expressions did not significantly differ between the first and second weeks for the Vit. C-p and 10^−10^, 10^−8^, and 10^−7^ M calcitriol groups (Table 2); Vit. C-p did not affect OCN mRNA expression in either week. However, the 10^−8^and 10^−7^ M calcitriol groups displayed a twofold increase in OCN mRNA expression at both weeks compared with that of the control and Vit. C-p groups (Figure 3(e) and Supplemental Table 5).

### 3.8. Vitamin D Receptor mRNA Expression at the First and Second Weeks

All tested groups presented nonsignificant VDR mRNA expression changes between the first and second weeks (*P* > 0.05, Table 2). Only the 10^−7^ M calcitriol group revealed a significant VDR mRNA expression, which was 2.4 times higher than that of other groups, including the Vit. C-p group at both weeks (Figure 3(f) and Supplemental Table 6).

### 3.9. ALP Enzyme Activity Assay

mRNA examination revealed that the decisive calcitriol fold changes were associated with 10^−8^ M and 10^−7^ M calcitriol; therefore, 10^−8^ M and 10^−7^ M calcitriol were used to examine the ALP activity (activity/min/mg protein) at the second and third weeks. The fold change of osteogenesis-related ALP activity was assessed in the control, Vit. C-p, 10^−8^ M calcitriol, and 10^−7^ M calcitriol groups (Table 3).

**Figure 4:**
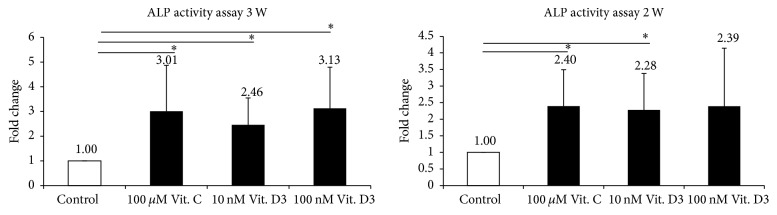
Alkaline phosphatase activity assay of osteoblast differentiation for second and third weeks.

Time studies revealed nonsignificant variation in the fold change of ALP activity between the second and third weeks (Figure 4). Compared with that in the control group, fold change in the Vit. C-p, 10^−8^ M calcitriol, and 10^−7^ M calcitriol groups increased by 2.40 ± 1.10 (*P* = 0.021), 2.28 ± 1.10 (*P* = 0.0033), and 2.39 ± 1.75 (*P* = 0.114) times, respectively, in the second week and 3.01 ± 1.86 (*P* = 0.042), 2.46 ± 1.09 (*P* = 0.018), and 3.13 ± 1.66 (*P* = 0.023) times, respectively, in the third week. These results revealed that 10^−8^ M calcitriol and Vit. C-p significantly enhanced the fold change of ALP activity at the second and third weeks.

## 4. Discussion

Tissue engineering by using MSCs is a recent therapeutic modality. MSCs, multipotent cells differentiating into various functional mesodermal tissue cell types [19], compose several tissues types and can differentiate into various functional cells, including osteoblasts. The periosteum serves as a niche for progenitor cells and a rich vasculature supply for the bone it envelops [20]. The proliferation rate of the periosteal cells was higher than that of marrow stromal cells [21], and P-MSCs were ideal candidates for bone tissue regeneration [22]. Furthermore, P-MSCs are considered useful for reducing the cell culture time, thus reducing both cost and contamination risk [21].

In this study, the osteogenic, chondrogenic, and adipogenic differentiation of PDCs was induced under specific differentiation media. This observation was in concordance with that described in previous studies evaluating cells extracted from the gingival tissue and bone marrow [23]. The present study results comply with the minimal standards required for the phenotypic and functional definition of human MSCs, as stated by the International Society for Cellular Therapy [1]. The results also reveal that P-MSCs can be isolated and expanded, revealing characteristics similar to those typically described for bone marrow stromal cells.

Bone MSCs exhibited osteogenic differentiation with dexamethasone, ascorbate, and *β*-glycerophosphate [24]. Calcitriol in combination with ascorbic acid promotes the differentiation of human MSCs into osteoblasts [25]. Furthermore, calcitriol is crucial for calcium homeostasis and bone metabolism; it influences the cardiovascular system, but the underlying mechanisms remain unclear [26]. Therefore, dexamethasone, ascorbate, and *β*-glycerophosphate were included in this study for enhancing P-MSC differentiation. Moreover, according to our review of relevant literature, this is the first study revealing the osteogenic potential of P-MSCs using different calcitriol concentrations by comparing the osteogenic gene expression.

Human MSC trials using early passage cellular cultures may avoid the senescent effects of expansion and reduce the cell culture period, thus reducing both cost and contamination risk. In accordance with previous osteogenic differentiation study [26], this study examined P-MSCs at passages 3–5 and subsequent cultures for 2 weeks under controlled conditions.

Calcitriol induced the expression of both early [6] and late-stage markers of osteoblast differentiation in MSCs including ALP, osteopontin, BSP, OCN mRNAs [24], and matrix Gla protein in osteoblast-like cell cultures [27]. The effects of varying calcitriol concentrations on osteogenesis have not been extensively discussed. Therefore, ascorbic acid and 10^−7^, 10^−8^, 10^−9^, and 10^−10^ M calcitriol concentrations regulated the ALP activity and osteogenic gene expression of ALP, BSP, CBFA1, Col-1, OCN, and VDR mRNAs.

Vit. C-p considerably regulated the ALP and CBFA1 mRNA expression and ALP activity at the first and second weeks as well as BSP and Col-1 mRNA expression at the first week. The 10^−10^ M calcitriol group revealed some changes in BSP and Col-1 mRNA expression at the first and second weeks, respectively (*P* < 0.05, Supplemental Tables 2 and 4). The 10^−9^ M calcitriol group mainly influenced ALP, Col-1, and OCN mRNA expression at both weeks and BSP mRNA expression at the first week (*P* < 0.05, Supplemental Tables 1–6). Furthermore, 10^−8^ M calcitriol majorly stimulated ALP, CBFA1, Col-1, and OCN mRNA expression at both weeks and BSP mRNA expression at the first week (*P* < 0.05, Supplemental Tables 1–6). A concentration of 10^−7^ M calcitriol intensified the fold change of ALP, BSP, OCN, Col-1, and VDR mRNA expression at both weeks as well as the CBFA1 mRNA expression at the second week (*P* < 0.05, Supplemental Tables 1–6).

ALP, BSP, and CBFA1 mRNA expression was negatively correlated between the 10^−7^, 10^−8^, and 10^−9^ M calcitriol groups and the Vit. C-p group at both weeks. However, the 10^−7^ M calcitriol group revealed a higher expression than did the Vit. C-p group with respect to OCN and VDR mRNA expression at both weeks and Col-1 mRNA expression at the second week. By contrast, the OCN mRNA expression at both weeks was higher in the 10^−8^ M calcitriol group than in the Vit. C-p group. More trials are required to elucidate the effect of calcitriol on osteogenic induction.

Dose studies revealed that 10^−7^ M calcitriol, and not other examined calcitriol concentrations, highly enhanced VDR mRNA expression at both tested weeks; moreover, compared with 10^−8^ M calcitriol, 10^−7^ M calcitriol weakly expressed ALP mRNA. After time studies revealed nonsignificant differences between the first and second weeks, we applied the average values of both weeks for analyzing the developing patterns (Supplemental Tables 1–6). Therefore, the 10^−7^and 10^−8^ M calcitriol groups exhibited higher ALP, CBFA1, and OCN mRNA expression than did the 10^−9^ M calcitriol group at both weeks; a similar effect was observed for Col-1 mRNA expression at the second week.

ALP, OCN, and Col-1 are markers capable of detecting early osteogenic cell differentiation [28]; by contrast, ALP acts as an ectoenzyme in the degradation of inorganic pyrophosphate for releasing phosphate for mineralization [18]. OCN, a major noncollagen bone protein, is a crucial osteogenic marker regulating the formation of mineral nodules, causing osteogenesis [29]. Col-1 is the most abundant matrix protein and OCN regulates matrix synthesis [30]. Both OCN and Col-1 are acidic bone matrix proteins with crucial roles in matrix synthesis and are characteristic of mature matrix-forming osteoblasts. However, OCN, osteopontin, and BSP expression are considered the mature osteogenic markers [28, 31, 32]. Calcitriol significantly upregulated OCN and Col-1 mRNA expression [14, 25, 33] and did not substantially affect CBFA1 or ALP gene expression [14]. However, our results revealed that 10^−7^and 10^−8^ M calcitriol upregulated osteogenesis by increasing ALP, BSP, CBFA1, Col-1, and OCN mRNA expression at both weeks. Moreover, VDR, a hormone-regulated transcription factor that interacts with coactivators and corepressors, is associated with chromatin histone modifications and transcriptional regulation [34]. Similar to a previous study stating that calcitriol increases VDR mRNA [14]. We confirmed that 10^−7^ M calcitriol significantly stimulates VDR mRNA expression.

According to the National Institutes of Health, 20–50 ng/mL (50–125 nmol/L) of serum 25-hydroxyvitamin D is desirable, but concentrations exceeding 30 ng/mL (75 nmol/L) are not consistently associated with an increased benefit. Serum 25-hydroxyvitamin D levels exceeding 50 ng/mL (125 nmol/L) may cause complications. However, this study indicated that 10^*‒*7^ M, which equals 100 nmol/L calcitriol, significantly upregulates VDR mRNA. Additional studies are required to explore the advantages and disadvantages of high calcitriol concentrations.

In contrast to a previous study which reported that ALP activity was inhibited by tumor-growing factor-beta (0.1–10 ng/mL) and increased by calcitriol (50 nM, which equals 5 × 10^−8^ M) [33], our results revealed that 10^−7^ and 10^−8^ M calcitriol and Vit. C-p enhanced P-MSC-related ALP activity.

The current results clearly demonstrate the feasibility of isolating MSCs from the periosteum and differentiating them into osteoblasts, chondrocytes, and adipocytes. Alveolar periosteum is rich in MSCs, which can be easily obtained from routine oral surgery and poses fewer complications; thus, they are suitable candidate donor sites for tissue engineering.

This time- and dose-based study assumed that Vit. C-p and 10^−7^and 10^−8^ M calcitriol share some mechanisms for inducing osteogenesis by increasing the ALP activity and ALP, BSP, CBFA1, and Col-1 mRNA expression. Moreover, 10^−8^ and 10^−7^ M calcitriol have more potential osteoinductivity than does 10^−9^ M calcitriol on alveolar P-MSCs. Calcitriol exclusively induces OCN and VDR mRNA osteoinduction.

Consistent with the results of some previous MSC studies, Vit. C-p and calcitriol significantly enhanced BSP mRNA at the first week; however, only 10^−7^ M calcitriol significantly upregulated BSP mRNA at the second week in this study. The small sample size and the nature of the P-MSCs possibly played a role on this variation. In addition, the nonsignificant difference between the first 2 weeks can be partially explained by the fact that BSP is considered a mature osteoblast marker. In the future, we plan to increase the tested sample number and evaluate the P-MSC cultures for changes in BSP mRNA at later stages of in vitro differentiation and maturation.

## 5. Conclusion

In summary, alveolar periosteum is rich in MSCs. Human P-MSCs can be induced to proliferate and differentiate into an osteogenic lineage. There is a trend showing that P-MSCs treated with Vit. C-p and 10^−9^, 10^−8^, and 10^−7^ M calcitriol promote ALP activity as well as the osteogenic differentiation of ALP, BSP, CBFA1, and Col-1 mRNA expression. By contrast, 10^−8^and 10^−7^ M calcitriol enhanced osteogenic OCN mRNA expression significantly more than did Vit. C-p. The 10^−7^ M calcitriol group exhibited upregulated VDR mRNA expression. Further investigation is required into the significance of the osteoinductive mechanism between Vit. C-p and calcitriol.

## Supplementary Material

ALP mRNA fold-change corresponding to calcitriol time and dose studies.

## Figures and Tables

**Table 1:** List of primers used in this study.

Gene	Primer design
ALP	Hs01029144_m1
BSP	Forward primer 5′-AAAGTGAGAACGGGGAACCT-3′
	Reverse primer 5′-GATGCAAAGCCAGAATGGAT-3′
CBFA1	Hs00231692_m1
Col-1	Forward primer 5′-CCTCAAGGGCTCCAACGAG-3′
	Reverse primer 5′-TCAATCACTGTCTTGCCCCA-3′
OCN	Forward primer 5′-GTGCAGCCTTTGTGTCCAAG-3′
	Reverse primer 5′-GTCAGCCAACTCGTCACAGT-3′
VDR	Hs01045846_m1
GAPDH	Hs99999905_m1

**Table 2:** Fold change significance of tested mRNAs (first week versus second week).

Group	mRNA					
	ALP	BSP	CBFA1	Col-1	OCN	VDR
Vit. C	0.445	0.091	0.898	0.980	0.925	0.432
10^−10^ MD	0.857	0.624	0.505	0.044^*∗*^	0.303	0.900
10^−9^ MD	0.330	0.102	0.421	0.038^*∗*^	0.043^*∗*^	0.235
10^−8^ MD	0.813	0.341	0.535	0.143	0.473	0.321
10^−7^ MD	0.017^*∗*^	0.529	0.009^*∗∗*^	0.067	0.871	0.517

^*∗*^ *p* < 0.05 and ^*∗∗*^ *p* < 0.01.

Exploring the statistical significance of the tested mRNAs' fold change between the first and second weeks and determining if the mRNAs could be discussed by combining the two sets of data.

**Table 3:** Fold change of ALP activity assays corresponding to calcitriol dose studies at the second and third weeks.

	Time points	Fold change mean ± SD	Vit. C	*E* − 8MD	*E* − 7MD
Control	2 W		0.02^*∗*^	0.03^*∗*^	0.11
	3 W		0.04^*∗*^	0.02^*∗*^	0.02^*∗*^
Vit. C	2 W	2.4 ± 1.10		0.88	1.00
	3 W	3.01 ± 1.86		0.62	0.92
*E* − 8MD	2 W	2.28 ± 1.10			0.91
	3 W	2.46 ± 1.09			0.53
*E* − 7MD	2 W	2.39 ± 1.75			
	3 W	3.13 ± 1.66			

^*∗*^ *p* < 0.05.

*E* − 8MD: 10^−8^ M calcitriol; *E* − 7MD: 10^−7^ M calcitriol; Vit. C: ascorbic acid (100 *µ*M).
