# Supplementary material for: Potential Osteoinductive Effects of Calcitriol on the m-RNA of Mesenchymal Stem Cells Derived from Human Alveolar Periosteum
Source: Biomed Res Int. 2016 Dec 25;2016:3529561. doi: 10.1155/2016/3529561 (PMC5220409; doi:10.1155/2016/3529561)
Supplement: Supplementary file 1 — ALP mRNA fold-change corresponding to calcitriol time and dose studies. [file 3529561.f1.docx]

**Supplemental Table 1.** ALP mRNA fold-change corresponding to calcitriol time and dose studies

| ALP | Time | mean±SD | Vit.C | E-10MD | E-9MD | E-8MD | E-7MD |
| --- | --- | --- | --- | --- | --- | --- | --- |
| Control | 1W | 1 | 0.028* | 0.291 | 0.012* | 0.002** | 0.020* |
|  | 2W | 1 | 0.025* | 0.586 | 0.009** | 0.008** | 0.001** |
| Vit.C | 1W | 3.68±2.24 |  | 0.025* | 0.074 | 0.532 | 0.155 |
|  | 2W | 5.12±3.34 |  | 0.023* | 0.068 | 0.875 | 0.899 |
| E-10MD | 1W | 0.93±0.14 |  |  | 0.009** | 0.002** | 0.020* |
|  | 2W | 0.95±0.21 |  |  | 0.009** | 0.008** | 0.001** |
| E-9MD | 1W | 1.59±0.41 |  |  |  | 0.006** | 0.753 |
|  | 2W | 1.93±0.61 |  |  |  | 0.033* | 0.006** |
| E-8MD | 1W | 4.50±1.69 |  |  |  |  | 0.026* |
|  | 2W | 4.82±2.44 |  |  |  |  | 0.969 |
| E-7MD | 1W | 1.49±0.37 |  |  |  |  |  |
|  | 2W | 4.88±1.61 |  |  |  |  |  |

*p* < 0.05: *, *p* < 0.01: **

Abbreviations: 1W: 1-week culture; 2W: 2-week culture; 1+2W: combined 1- and 2-week data that were discussed when nonsignificance existed; E-7MD: 10^−7^ M calcitriol; E-8MD: 10^−8^ M calcitriol; E-9MD: 10^−9^ M calcitriol; E-10MD: 10^−10^ M calcitriol; Vit.C: ascorbic acid (100 µM).

**Supplemental Table 2.** BSP mRNA fold-change corresponding to calcitriol time and dose studies

| BSP | Time | mean±SD | Vit.C | E-10MD | E-9MD | E-8MD | E-7MD |
| --- | --- | --- | --- | --- | --- | --- | --- |
| Control | 1W | 1 | 0.001** | 0.036* | 0.026* | 0.009** | 0.023* |
|  | 2W | 1 | 0.255 | 0.289 | 0.245 | 0.095 | 0.031* |
| Vit.C | 1W | 2.34±0.59 |  | 0.052 | 0.374 | 0.812 | 0.879 |
|  | 2W | 1.42±0.77 |  | 0.856 | 0.605 | 0.536 | 0.369 |
| E-10MD | 1W | 1.52±0.46 |  |  | 0.356 | 0.086 | 0.179 |
|  | 2W | 1.34±0.66 |  |  | 0.730 | 0.421 | 0.260 |
| E-9MD | 1W | 1.91±0.74 |  |  |  | 0.342 | 0.555 |
|  | 2W | 1.21±0.38 |  |  |  | 0.244 | 0.116 |
| E-8MD | 1W | 2.48±0.95 |  |  |  |  | 0.738 |
|  | 2W | 1.79±0.93 |  |  |  |  | 0.876 |
| E-7MD | 1W | 2.25±1.00 |  |  |  |  |  |
|  | 2W | 1.88±0.75 |  |  |  |  |  |

*p* < 0.05: *, *p* < 0.01: **

Abbreviations: E-7MD: 10^−7^ M calcitriol; E-8MD: 10^−8^ M calcitriol; E-9MD: 10^−9^ M calcitriol; E-10MD: 10^−10^ M calcitriol; Vit.C: ascorbic acid (100 µM).

**Supplemental Table 3.** CBFA1 mRNA fold-change corresponding to calcitriol time and dose studies

| CBFA1 | Time | mean±SD | Vit.C | E-10MD | E-9MD | E-8MD | E-7MD |
| --- | --- | --- | --- | --- | --- | --- | --- |
| Control | 1W | 1 | 0.036* | 0.633 | 0.769 | 0.011* | 0.129 |
|  | 2W | 1 | 0.014* | 0.096 | 0.176 | 0.030* | <0.001*** |
| Vit.C | 1W | 1.42±0.37 |  | 0.075 | 0.057 | 0.801 | 0.263 |
|  | 2W | 1.45±0.32 |  | 0.060 | 0.061 | 0.476 | 0.118 |
| E-10MD | 1W | 1.04±0.18 |  |  | 0.855 | 0.030* | 0.387 |
|  | 2W | 1.11±0.13 |  |  | 0.928 | 0.065 | 0.001** |
| E-9MD | 1W | 1.02±0.15 |  |  |  | 0.021* | 0.281 |
|  | 2W | 1.10±0.15 |  |  |  | 0.064 | 0.002** |
| E-8MD | 1W | 1.48±0.32 |  |  |  |  | 0.139 |
|  | 2W | 1.67±0.57 |  |  |  |  | 0.685 |
| E-7MD | 1W | 1.16±0.22 |  |  |  |  |  |
|  | 2W | 1.79±0.29 |  |  |  |  |  |

*p* < 0.05: *, *p*< 0.01: **, *p*< 0.001***

Abbreviations: E-7MD: 10^−7^ M calcitriol; E-8MD: 10^−8^ M calcitriol; E-9MD: 10^−9^ M calcitriol; E-10MD: 10^−10^ M calcitriol; Vit.C: ascorbic acid (100 µM).

**Supplemental Table 4.** Col-1 mRNA fold-change corresponding to calcitriol time and dose studies

| COL-1 | Time | mean±SD | Vit.C | E-10MD | E-9MD | E-8MD | E-7MD |
| --- | --- | --- | --- | --- | --- | --- | --- |
| Control | 1W | 1 | 0.014* | 0.805 | 0.013* | 0.001** | 0.027* |
|  | 2W | 1 | 0.090 | 0.023* | 0.001** | 0.014* | 0.010* |
| Vit.C | 1W | 1.91±0.66 |  | 0.016* | 0.045* | 0.914 | 0.910 |
|  | 2W | 1.90±1.05 |  | 0.293 | 0.381 | 0.186 | 0.042* |
| E-10MD | 1W | 0.98±0.22 |  |  | 0.087 | 0.002** | 0.030* |
|  | 2W | 1.36±0.28 |  |  | 0.506 | 0.034* | 0.016* |
| E-9MD | 1W | 1.20±0.14 |  |  |  | 0.006 | 0.068 |
|  | 2W | 1.46±0.19 |  |  |  | 0.042* | 0.018* |
| E-8MD | 1W | 1.95±0.43 |  |  |  |  | 0.967 |
|  | 2W | 3.07±1.48 |  |  |  |  | 0.198 |
| E-7MD | 1W | 1.97±0.80 |  |  |  |  |  |
|  | 2W | 4.98±2.65 |  |  |  |  |  |

*p* < 0.05: *, *p* < 0.01: **

Abbreviations: E-7MD: 10^−7^ M calcitriol; E-8MD: 10^−8^ M calcitriol; E-9MD: 10^−9^ M calcitriol; E-10MD: 10^−10^ M calcitriol; Vit.C: ascorbic acid (100 µM).

**Supplemental Table 5.** OCN mRNA fold-change corresponding to calcitriol time and dose studies

| OCN | Time | mean±SD | Vit.C | E-10MD | E-9MD | E-8MD | E-7MD |
| --- | --- | --- | --- | --- | --- | --- | --- |
| Control | 1W | 1 | 0.262 | 0.892 | 0.001** | 0.002** | 0.006** |
|  | 2W | 1 | 0.097 | 0.157 | 0.002** | 0.001** | 0.031* |
| Vit.C | 1W | 1.11±0.21 |  | 0.516 | 0.860 | 0.012* | 0.012* |
|  | 2W | 1.13±0.15 |  | 0.607 | 0.204 | 0.004** | 0.047* |
| E-10MD | 1W | 1.02±0.25 |  |  | 0.552 | 0.008** | 0.009** |
|  | 2W | 1.20±0.29 |  |  | 0.759 | 0.014* | 0.065 |
| E-9MD | 1W | 1.09±0.04 |  |  |  | 0.011* | 0.019* |
|  | 2W | 1.26±0.12 |  |  |  | 0.018* | 0.105 |
| E-8MD | 1W | 1.79±0.38 |  |  |  |  | 0.307 |
|  | 2W | 2.01±0.46 |  |  |  |  | 0.603 |
| E-7MD | 1W | 2.24±0.71 |  |  |  |  |  |
|  | 2W | 2.35±1.14 |  |  |  |  |  |

*p* < 0.05: *, *p* < 0.01: **

Abbreviations: E-7MD: 10^−7^ M calcitriol; E-8MD: 10^−8^ M calcitriol; E-9MD: 10^−9^ M calcitriol; E-10MD: 10^−10^ M calcitriol; Vit.C: ascorbic acid (100 µM).

**Supplemental Table 6.** VDR mRNA fold-change corresponding to calcitriol time and dose studies

| VDR | Time | mean±SD | Vit.C | E-10MD | E-9MD | E-8MD | E-7MD |
| --- | --- | --- | --- | --- | --- | --- | --- |
| Control | 1W | 1 | 0.940 | 0.813 | 0.219 | 0.204 | 0.001** |
|  | 2W | 1 | 0.307 | 0.274 | 0.868 | 0.960 | 0.004** |
| Vit.C | 1W | 1.01±0.14 |  | 0.808 | 0.291 | 0.279 | 0.001** |
|  | 2W | 0.93±0.15 |  | 0.712 | 0.342 | 0.512 | 0.004** |
| E-10MD | 1W | 0.97±0.25 |  |  | 0.532 | 0.280 | 0.001** |
|  | 2W | 0.96±0.08 |  |  | 0.396 | 0.637 | 0.004** |
| E-9MD | 1W | 0.88±0.20 |  |  |  | 0.092 | <0.001*** |
|  | 2W | 1.01±0.09 |  |  |  | 0.980 | 0.005** |
| E-8MD | 1W | 1.16±0.25 |  |  |  |  | 0.001** |
|  | 2W | 1.00±0.20 |  |  |  |  | 0.006** |
| E-7MD | 1W | 2.72±0.70 |  |  |  |  |  |
|  | 2W | 2.37±0.72 |  |  |  |  |  |

*p* < 0.05: *, *p* < 0.01: **, *p* < 0.001***

Abbreviations: E-7MD: 10^−7^ M calcitriol; E-8MD: 10^−8^ M calcitriol; E-9MD: 10^−9^ M calcitriol; E-10MD: 10^−10^ M calcitriol; Vit.C: ascorbic acid (100 µM).
